# Draft Genome Sequence of a Sulfur-Oxidizing Bacterium, Aquamicrobium lusatiense NLF2-7, Isolated from Livestock Wastewater

**DOI:** 10.1128/mra.00799-22

**Published:** 2022-12-21

**Authors:** Sang Eun Jeong, Kook-Il Han, Ji Young Jung, Mi-Hwa Lee, Jong-Guk Kim, Hyun Mi Jin

**Affiliations:** a Nakdonggang National Institute of Biological Resources, Sangju, Republic of Korea; b School of Applied Biosciences, Kyungpook National University, Daegu, Republic of Korea; Loyola University Chicago

## Abstract

Here, we report the draft genome sequence of Aquamicrobium lusatiense NLF2-7, a Gram-negative, aerobic, non-flagellum-forming, rod-shaped bacterium that was isolated from livestock wastewater in South Korea. The assembled genome sequence is 5,201,486 bp, with 4,972 protein-coding sequences in 12 contigs, and possess the genes for the sulfur oxidation pathway.

## ANNOUNCEMENT

The species Aquamicrobium lusatiense, a member of the family *Phyllobacteriaceae* of the class *Alphaproteobacteria*, was reclassified from Defluvibacter lusatiensis (1) by Kämpfer et al. (2). Interestingly, most species of the genus *Aquamicrobium* have been isolated from pollutant-loaded environments, such as wastewater treatment plants (WWTPs) (2), activated sewage sludge (3), biofilters (4), a crude-oil-contaminated tidal flat (5), and chlorobenzoate-contaminated soil (6). The genomic sequence of strain NLF2-7 will provide insight into the sulfur oxidation pathways of *Aquamicrobium* species.

Strain NLF2-7 was isolated from the biofilm of a sample collected from a livestock WWTP in Nonsan, Republic of Korea. A liquid sample from the WWTP was serially diluted in phosphate-buffered saline (PBS). One of the problems in WWTPs is the increase in emission of odor pollutants such as hydrogen sulfide, which can cause damage to the health of human populations and ecosystems. To control emissions of this gas, sulfur-oxidizing bacteria can be used to convert hydrogen sulfide to sulfate (7). Therefore, in this study, sulfur oxidation bacterial medium (8) was used for isolation of strain NLF2-7. After 10 days of incubation at 30°C, colonies were selected and incubated in fresh medium. The 16S rRNA genes of the colonies were PCR amplified using the universal F1 and R13 primers, and the resulting sequences were compared with 16S rRNA gene sequences of all type strains using the Nucleotide Similarity Search tool.

Genomic DNA was extracted, using the Maxwell 16 DNA purification kit (Promega, USA) according to the manufacturer’s instructions, from a plate culture that had been grown at 30°C for 5 days in Trypticase soy agar (Difco, USA). The genome of Aquamicrobium lusatiense NLF2-7 was sequenced with a Pacific Biosciences (PacBio) RS II sequencer using a 15-kb SMRTbell template library and with an Illumina HiSeq X Ten sequencer using 151-bp paired-end reads, at Theragen Bio (Suwon, South Korea). A size selection cutoff value of 7,000 bp was used. The size-selected SMRTbell library was annealed and bound according to the single-molecule real-time (SMRT) Link setup and sequenced on a PacBio instrument. The SMRTbell template preparation kit v2.0 (PacBio) and TruSeq Nano DNA library preparation kit (Illumina) were used to prepare sequencing libraries. The 94,823 total reads (*N*_50_, 11,631 bp) produced by the PacBio RS II platform were assembled, whereas the Illumina platform produced 11,926,744 total reads. Then, *de novo* assembly, error correction, and annotation were performed with Canu v1.7 (9), Pilon v1.22 (10), and Prokka v1.1.0 (11), respectively. Default parameters were used for all software unless otherwise specified. The assembled genome has a length of 5,201,486 bp, with 12 contigs and an average GC content of 62.30%.

Genome features of Aquamicrobium lusatiense strain NLF2-7 are presented in Table 1. Genome annotation was performed using the Prokaryotic Genome Annotation Pipeline (PGAP) v6.1 (12). Based on the PGAP results, the draft genome sequence of A. lusatiense NLF2-7 has 58 RNAs (48 tRNAs, 6 rRNAs, and 4 noncoding RNA), 4,839 protein-coding genes, and 115 pseudogenes. The genome of strain NLF2-7 contains a complete Sox pathway (i.e., *soxXYZABCD*), which potentially allows the oxidation of both sulfur atoms of thiosulfate completely to sulfate.

**TABLE 1:** Genome features of Aquamicrobium lusatiense NLF2-7

Genome feature	Value
Genome size (bp)	5,201,486
GC content (%)	62.30
No. of rRNAs (5S, 16S, 23S)	6 (2, 2, 2)
No. of tRNAs	48
No. of noncoding RNAs	4
No. of protein-coding genes	4,839
No. of pseudogenes	115

### Data availability.

This whole-genome shotgun project has been deposited in DDBJ/ENA/GenBank under the accession number JAKSED000000000, BioProject accession number PRJNA807442, and BioSample accession number SAMN25981959. The raw reads have been deposited in the Sequence Read Archive (SRA) under SRA accession numbers SRR18547513 and SRR22071623.
